# *Alatococcus*, a new genus of Sapindaceae from Espirito Santo, Brazil

**DOI:** 10.3897/phytokeys.10.2718

**Published:** 2012-03-09

**Authors:** Pedro Acevedo-Rodríguez

**Affiliations:** 1 Department of Botany, MRC-166, Smithsonian Institution, P.O. Box 37012, Washington D.C. 20013-7012, USA

**Keywords:** *Alatococcus*, *Scyphonychium*, Sapindaceae, Espirito Santo, Brazil

## Abstract

The new genus *Alatococcus* (Sapindaceae) is described from Espirito Santo, Brazil. *Alatococcus* seems to be closely related to *Scyphonychium* of northeastern Brazil, as they both share similar vegetative morphology, flowers with zygomorphic calyx, fruits with indehiscent mericarps, and isopolar, tricolporate pollen grains. They however differ by the shape of the nectary disc, petal appendages, mericarps, and pollen shape and ornamentation. The new species *Alatococcus siqueirae* is described and illustrated.

## Introduction

The genus *Alatococcus* is herein described to accommodate a species that although similar to *Scyphonychium* by its zygomorphic calyx and indehiscent mericarps, differs by key floral, fruit, and pollen characters. The mono-specific genus *Scyphonychium* was described and placed in the tribe Cupanieae by Radlkofer in 1879 and differentiated from *Cupania*, *Vouarana*, and *Dilodendron* by its zygomorphic calyx, bilobed petal appendages (forming a pocket with the petal), and 2-carpellate ovaries. Later in 1989, Ferrucci transferred *Scyphonychium* into the tribe Sapindoideae after describing its fruits as schizocarpic, containing one or two woody, subglobose mericarps, and its seeds as semispherical and exarillate. *Alatococcus* is herein placed into the Sapindoideae because of its indehiscent mericarps and although similar to *Scyphonychium*, it can be differentiated by the following key.

### Key to Alatococcus and Scyphonychium

**Table:** 

1	Flowers in sub-cincinni; nectary disc semi-annular; petal appendages hood-shaped, with corniform crest and wooly ligule; ovary 3-carpellate; fruits of a single, winged mericarp; pollen subspheroidal, striate	*Alatococcus*
–	Flowers in dichasia; nectary disc cupular; petal appendages simple and bilobed, connate to petal margins to form a pocket; ovary 2-carpellate; fruit of 1 or 2, unwinged mericarps; pollen oblate, perforate	*Scyphonychium*

## Taxonomic treatment

### Alatococcus

Acev.-Rodr. gen. nov.

urn:lsid:ipni.org:names:77117837-1

http://species-id.net/wiki/Alatococcus

#### Remarks

Alatococcus siqueirae is distinguished by its fruits which consist of a single, woody, subglobose, mericarp with a wing that surrounds the entire longitudinal circumference of the locule.

#### Type.

*Alatococcus siqueirae* Acev.-Rodr.

#### Description.

 Small to medium-sized trees. Stipules wanting. Leaves alternate, paripinnate; leaflets entire; distal leaflet rudimentary. Inflorescences distal or axillary, paniculate thyrses, with flowers in lateral sub-cincinni. Flowers functionally unisexual; calyx zygomorphic, sepals 5, free, imbricate, the outer 2 smaller; petals 5, clawed, twice as long as the sepals; appendages hood-shaped, with corniform crest and wooly ligule; disc semi-annular; stamens 8; pollen isopolar, subspheroidal, colporate, with striate ornamentation; ovary 3-carpellate, with a single ovule per carpel; stigma shortly trifid. Fruit of a single, woody, sub-globose, winged mericarp, the wing surrounding the entire longitudinal circumference of the locule; seed solitary, exarillate with a large hilum at base.

#### Distribution.

 One species, known only from Espirito Santo, Brazil.

#### Etymology.

The name *Alatococcus* refers to the winged mericarps which characterizes the genus.

### Alatococcus siqueirae

Acev.-Rodr. sp. nov.

urn:lsid:ipni.org:names:77117838-1

http://species-id.net/wiki/Alatococcus_siqueirae

Fig. 1

#### Type.

**Figure 1.**
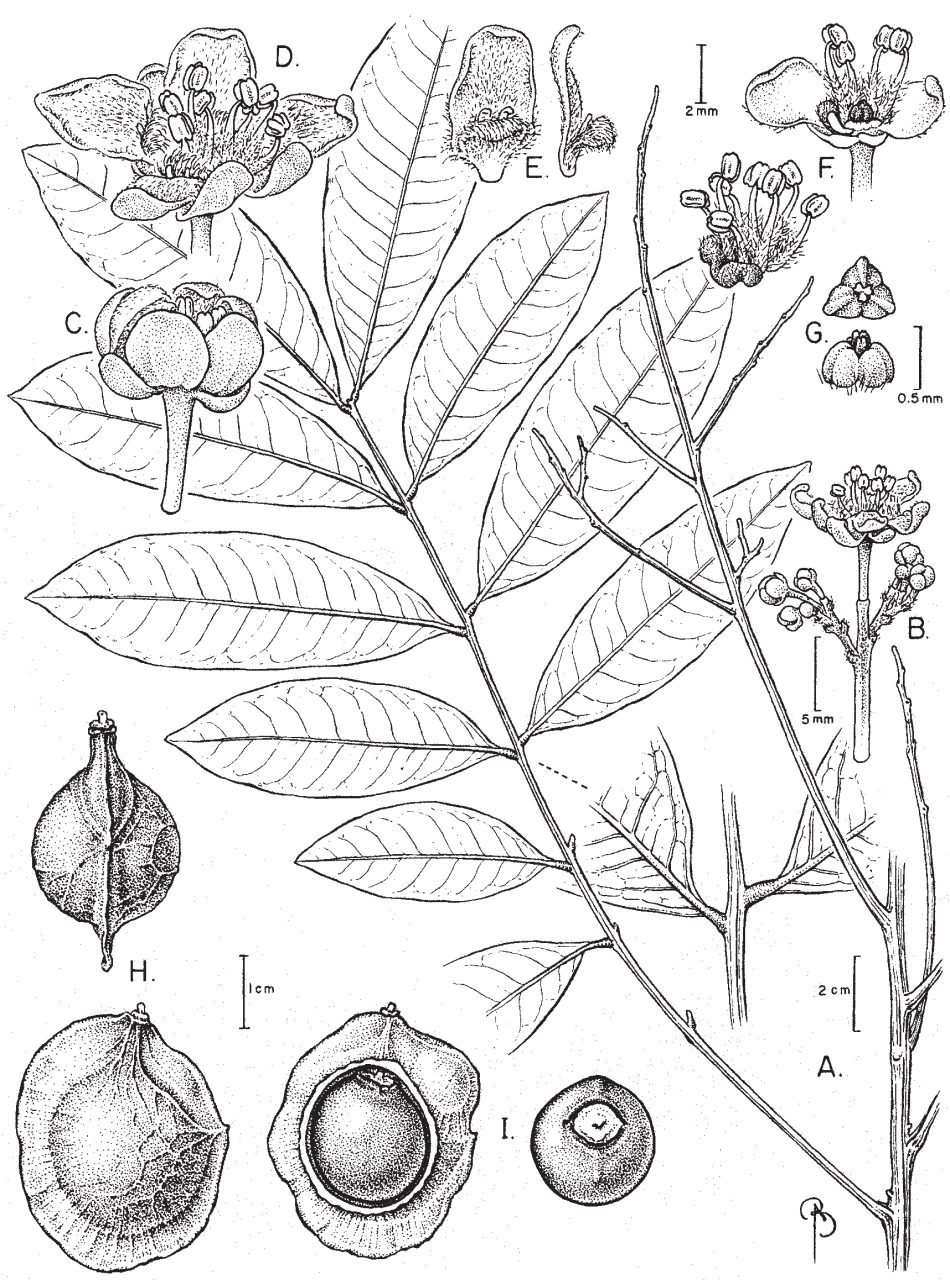
*Alatococcus siqueirae* **A** Branch with detail of petiolules **B** Sub-cincinnus **C** Staminate flower bud **D** Staminate flower **E** Petal with appendage, adaxial and lateral views **F** Portion of staminate flower showing unilateral nectary disc and stamens, staminate flower with removed petal showing nectary disc, stamens and pistillode **G** Pistillode, top and lateral views **H** Fruit, two lateral views **I** Dissected fruit showing wing, locule, mesocarp and seed, seed basal view. **A, H–I** from *Folli 1761* (K); **B–G** from *Siqueira and Magnago 639* (US).

 Brazil; Espirito Santo. Linhares, Povoaçao. Estrada Povaçao a Linhares, beira do rio; matas de cabrucas de cacao, mata de tabuleiro, 19°33'02"S, 39°50'40"W, 7 May 2011 (fl), *G.S. Siqueira & L.F. Silva Magnago 639* (holotype US!; isotypes CVRD, K!, NY!, RB!). Fig. 1.

#### Description.

 Tree to 14–19 m tall. Branches terete, glabrous, grayish with lines of lenticels. Leaves paripinnate; petiole plus rachis 20–32 cm long, flattened adaxially, minutely lenticellate; petiolules 5–12 mm long, pulvinate; leaflets 8–12, elliptic, oblong or nearly oblanceolate, 14–20.5 × 6–7.5 cm, chartaceous, glabrous, the base obtuse, sometimes slightly asymmetrical, the apex obtuse to acute, the margins entire, midvein and secondary veins abaxially prominent, lighter.

Thyrses axillary, to 60 cm long, axes appressed-pubescent to glabrous; flowers in sub-cincinni; pedicels 2.5–3 mm long, articulate at base. Calyx abaxially glabrous; sepals ovate, ciliate, outer sepals ca. 2.5 mm long, inner sepals ca. 3 mm long; petals ca. 6 mm long, lanceolate, clawed at base, obtuse at apex, abaxially sericeous; appendage hood-shaped, ca. 2 mm long, with a bi-corniform crest and a wooly ligule; nectary asymmetrical-pentagonous (2 lobes per petal except for the anterior petal that has no lobes or only slightly developed ones), pubescent; stamens of unequal lengths, 1.5–2.5 mm long, filament flattened, lanose on lower half, anthers ellipsoid; pistillode trigonous, tricarpellate. Fruit of one, subglobose, well-developed, winged mericarp and two rudimentary mericarps; mericarp woody, 3.5–4 × 2.5–3 cm, asymmetrical with style in lateral position; wing surrounding its longitudinal circumference; seeds exarillate, subglobose, ca. 1.7 cm long; hilum elliptic, ca. 7 mm wide.

#### Pollen.

**Figure 2.**
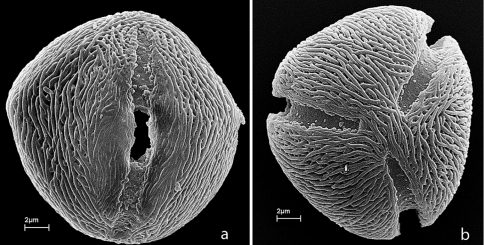
Pollen of *Alatococcus siqueirae*. **A** Equatorial view showing colpus and pore **B** Polar view showing three colpi and striate ornamentation. All from *Siqueira & Magnago 639* (US).

Pollen grains in *Alatococcus siqueirae* are isopolar, tricolporate, subspheroidal in equatorial view, trigonous in polar view, and with striate ornamentation (Fig 2a-b). Size as measured from 20 pollen grains using light microscopy is as follows: polar axis 20.11 µm (17.89–21.81µm); equatorial axis 19.16 µm (17.21–21.79 µm). Permanent pollen slides are deposited at Smithsonian’s pollen collection.

#### Vernacular names.

 baratinha, pitomba do rio doce.

**Distribution and ecology**. Known only from Espirito Santo, Brazil, on *tabuleiro*, gallery and tall forests.

#### Specimen examined.

 Brazil. Espirito Santo; Laranja da Terra, tall forest, 17 Dec 1992 (fr), *Folli 1761* (K, US), Linhares, road from Linhares to Fazenda Maria Bonita, gallery forest, 19°26'18"S, 39°58'00"W, 26 Oct 2010 (fr), *Folli 6734* (US).

#### Etymology.

 The epithet honors Geovani Siqueira, curator of the CVRD herbarium, who collected flowering material of the new species, allowing the determination to the generic level.

## Supplementary Material

XML Treatment for Alatococcus

XML Treatment for Alatococcus siqueirae
